# Primary tracheobronchial mucoepidermoid carcinoma - a retrospective study of 32 patients

**DOI:** 10.1186/1477-7819-11-62

**Published:** 2013-03-09

**Authors:** Zhengbo Song, Zhuo Liu, Jiwen Wang, Huineng Zhu, Yiping Zhang

**Affiliations:** 1 Department of Chemotherapy, Zhejiang Cancer Hospital, 38 GuangjiRoad, Gongshu District, Hangzhou 310022, P.R. of China; 2 Key Laboratory Diagnosis and Treatment Technology on Thoracic Oncology, 38 GuangjiRoad, Gongshu District, Hangzhou, Zhejiang Province 310022, P.R. of China; 3 Department of Oncological Surgery, Zhejiang Cancer Hospital, 38 GuangjiRoad, Gongshu District, Hangzhou 310022, P.R. of China; 4 Department of Pathology, Zhejiang Cancer Hospital, 38 GuangjiRoad, Gongshu District, Hangzhou 310022, P.R. of China

**Keywords:** Tracheobronchial, Mucoepidermoid carcinoma, Treatment, Prognosis

## Abstract

**Background:**

This retrospective study was designed to investigate the clinical characteristics, diagnosis, treatment and prognosis of primary tracheobronchial mucoepidermoid carcinoma (MEC).

**Methods:**

Clinical data were retrospectively analyzed from 32 patients with pathologically confirmed primary tracheobronchial MEC between January 1990 and December 2010 at Zhejiang Cancer Hospital. The Kaplan-Meier methods were used to estimate and compare survival rates.

**Results:**

There were 19 males and 13 females ranging in age from 7 to 73 years, with a median age of 28 years. Twenty-six of the 32 patients were treated with surgery alone. The other six patients were treated with surgery plus postoperative radiotherapy or chemotherapy. Six patients died during the follow-up time. The overall five-year survival rates were 81.25%, whereas the five-year survival rate of seven patients with high-grade tumors was only 28.6%. Stage I and II patients experienced better survival than Stage III and IV patients (the five-year survival rate was 100% and 43.6% respectively, *P*<0.001).

**Conclusions:**

Primary tracheobronchial MEC is a rare disease. Histologic grading and TNM (tumor-node-metastasis)staging are independent prognostic factors. Surgical resection is the primary treatment.

## Background

Primary mucoepidermoid carcinoma (MEC) of the tracheobronchial is an uncommon neoplasm, constituting only 0.1% to 0.2% of primary lung malignancies [1]. Its biological behavior and prognosis have not been well studied. Surgical resection is still the primary treatment [2,3]. This report reviews the clinical characteristics, diagnosis, treatment and prognosis of 32 cases with primary tracheobronchial MEC in our hospital from January 1990 to December 2010.

## Methods

### Patient eligibility

Patients who were receiving the treatment at our institution from January 1990 to December 2010 were included in this study. The Ethics Committee at Zhejiang Cancer Hospital approved the study. All patients were reviewed concerning their medical history and underwent physical examinations. The staging was performed for all patients according to the seventh TNM (tumor-node-metastasis) classification [4] in bronchial MEC. Bhattacharyya’s [5] staging system was adopted and used in tracheal MEC. The pathology was according to the World Health Organization Classification of Tumors (2004). Patient selection criteria are: (1) pathologically proven primary tracheobronchial mucoepidermoidcarcinoma; (2) all the patients were confirmed using chest computed tomography (CT), brain magnetic resonance imaging (MRI) and bone scan as well as ultrasound, and/or CT of the abdomen before surgery.

### Statistical analysis

The survival time was calculated from the start of treatment to the point of death or the last follow-up. The survival curves were calculated based on the method of Kaplan-Meier. Values of *P*<0.05 were considered significant. Analyses were conducted using the computer software SPSS version 16.0 (SPSS Inc., Chicago, IL, USA).

### Follow-up

After surgical intervention, patients were examined in the outpatient clinic at three-month intervals for the first two years and, thereafter, at six-month intervals. During follow-up periods, contrast enhanced CT scans of the chest were routinely performed at the same intervals as visits to the outpatient clinic. Follow-up data were obtained by retrospective review of the patient’s medical records and telephone surveys.

## Results

### Patient characteristics

Over the past 20 years, 8,310 patients were diagnosed with respiratory tumors in our hospital, and 32 of them were tracheobronchial primary MEC, which accounted for 0.38% of the whole population. This study group comprised 19 males and 13 females ranging in age from 7 to 73 years (median age, 28 years). According to the TNM staging system and Bhattacharyya’s staging system, the study included 10 Stage I, 8 Stage II, 11 Stage III, and 3 Stage IV patients (all 3of these patients were trachea MEC) (detailed in Table 1).

**Table 1:** Clinical characteristics of 32 patients

**Gender**	
**Male**	19
**Female**	13
**Age**	
**Range**	7 to 73
**Median**	28
**<30**	16
**≥30**	16
**Staging**	
**I**	10
**II**	8
**III**	11
**IV**	3
**Adjuvant treatment**	
**Yes**	6
**No**	26
**Grade**	
**High**	7
**Low**	25
**Tumor length**	
**≤3 cm**	17
**>3 cm**	15
**Surgical resection**	
**Radical**	28
**Palliative**	4
**Tumor location**	
**Tracheal**	13
**Bronchial**	19
**Lymphadenectomy**	
**Yes**	11
**No**	21
**Smoking history**	
**Yes**	5
**No**	27

### Treatment

All 32 patients underwent surgery, including 28 cases of radical excision and 4 cases of palliative resection. Thirteen tumors were located in tracheal and 19 in bronchial areas. Postoperative complications included one case of pneumonia. No patients died during treatment in the hospital. Twenty-seven patients who underwent operation were treated with surgery alone. There were 9 parenchyma-saving procedures (4 sleeve lobectomies, 1 upper and middle sleeve lobectomy, 2 main bronchus sleeve resection with end-to-end anastomosis, 2 main bronchus sleeve resection), 10 resection of anatomically related lung parenchyma (3 bilateral lobectomies, 6 lobectomies, 1 pneumonectomy) and 13 local tumor resections including 3 tumor resections plus carinaplasty, 4 single local tumor resection and 6 tracheal resection and end to end anastomosis. Among 32 patients, 11 were assigned to receive mediastinal lymphadenectomy, 21 without lymphadenectomy. Six patients were treated with surgery plus postoperative radiotherapy or chemotherapy. Chemotherapy was administered following resection in five of the seven high-grade MEC patients. The regimen comprisedNP (vinorelbine + cisplatin) regimen in four patients and DP (decetaxol + cisplatin) in one patient. One patient received postoperative radiotherapy for the positive resection margin.

### Follow-up and prognosis

**Figure 1:**
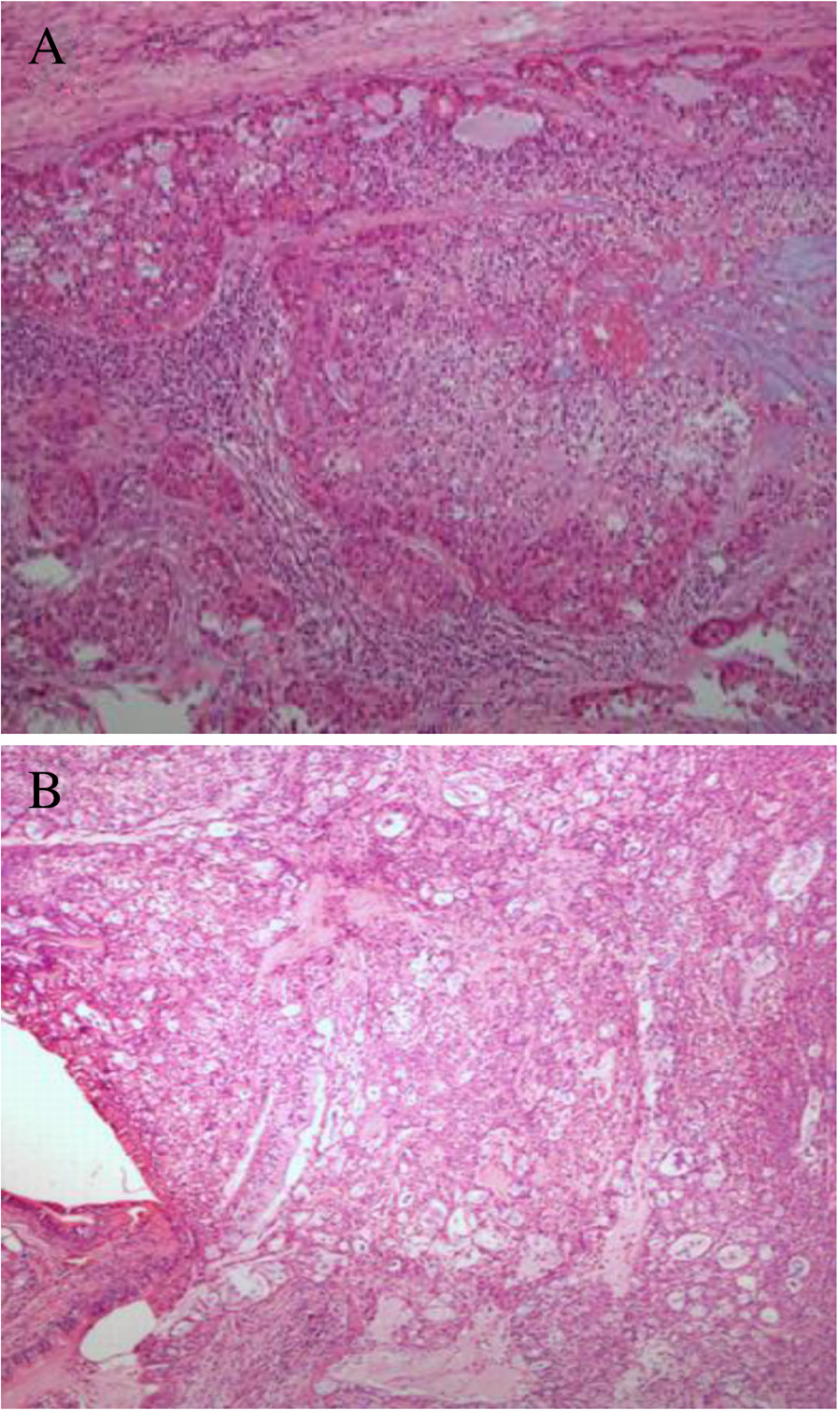
**(A) Tracheobronchial MEC with a high grade pathological diagnosis (HE; original magnification × 40).** (**B**) Tracheobronchial MEC with a low grade pathological diagnosis (HE; original magnification × 40).

**Figure 2:**
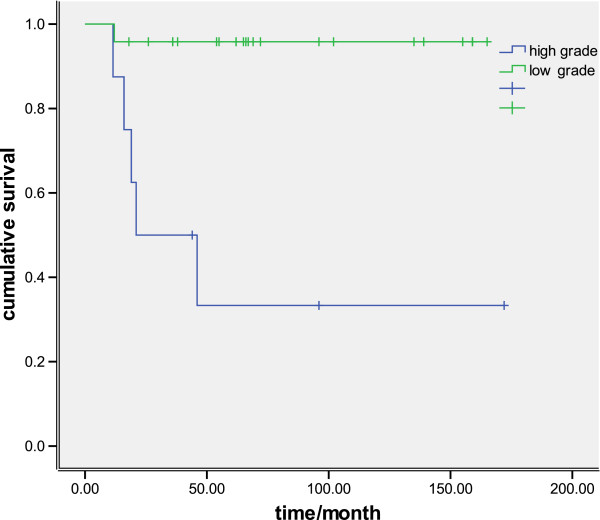
Kaplan-Meier curves comparing survival of patients with different histological grades.

**Figure 3:**
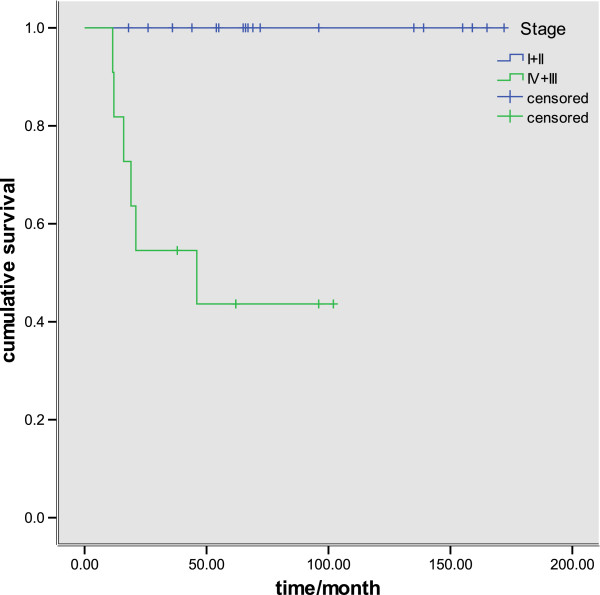
Kaplan-Meier curves comparing survival of patients with early TNM stage and late stage tumors.

Mean follow-up duration was 102 months (range 12 to 196). No patients were lost to follow-up. Six patients died during the follow-up. Five with high-grade histology and one with low-grade (Figure 1A, B). The overall five-year survival rates were 81.25%, whereas the five-year survival of seven patients with high-grade tumors was only 28.6% (Figure 2). The overall five-year survival rates were 100% compared with 43.6% between the Stage (I + II) and Stage (III + IV) patients, *P*<0.001) (Figure 3).

## Discussion

Primary tracheobronchial MEC is relatively uncommon, accounting for less than 1% of all cases of primary tracheobronchial carcinoma. The tumor was first described by Smetana *et al*.and Liebow*et al*. in 1952 [6,7]. Tracheobronchial MEC accounted for 0.38% of all pulmonary carcinomas at our institution. The frequency is similar to the previous reports.

Primary tracheobronchial MEC affects people of all ages and more than half of the patients were younger than 30 years [8-10]. In our series, 16 of the 32 patients were younger than 30 years, with a median age of 28 years in all of our patients (range of 7 to 73 years). A male predominance was described by many studies [11,12]. Nineteen cases were male and 13 were female in our series. The association with cigarette smoking was not notable [13,14], with only 5 of 32 patients in our study being current or ever smokers.

MEC of the trachea and bronchi are classified as low-grade or high-grade based on nuclear pleomorphism, mitotic activity and the presence or absence of necrosis [15]. The clinical behavior of tracheobronchial MEC has been reported to vary from low malignancy to highly malignant. Patients with low grade histology tend to behave in a low malignancy and do not require chemotherapy or radiotherapy with complete resection [14,15]. The present study also found significantly better survival in patients with histologically low-grade tumors than in patients with high-grade tumors.

TNM staging was a significant independent predictor of prognosis in patients with tracheobronchial MEC [2,16]. The five-year survival rates were 100% in Stage I and Stage II patients in our series; however, all of the six patients who died were Stage III and Stage IV patients.

Standard treatment for MEC is surgical resection [17]. Common surgical procedures include lobectomy, sleeve resection, local resection, segmental resection or endoscopic removal. It is difficult to remove tumors with an adequate margin in some cases, especially T4 tumors that are too large and are localized near important organs, so neo-chemotherapy may be an effective treatment.

Postoperative chemotherapy is not suggested for patients with low-grade MEC. Adjuvant chemotherapy or radiotherapy can be considered for patients with incomplete resection or advanced disease, but there is no strong evidence about their roles. In the present study, chemotherapy was administered following resection in five of the seven high-grade MEC patients; however, all of the patients had recurrence and four of them died during the follow-up.

## Conclusion

In summary, primary MEC of the tracheobronchial is a rare disease. Histological grade and TNM staging appear to be independent prognostic factors in our data. Surgical resection is the primary treatment. It is necessary to achieve further improvements in the clinical outcome of patients with such tumors by developing new therapeutic modalities.

## Abbreviations

CT: Computed tomography; DP: decetaxol + cisplatin; MEC: Mucoepidermoid carcinoma; MRI: Magnetic resonance imaging; NP: vinorelbine + cisplatin; TNM: Tumor-node-metastasis

## Competing interests

The authors declare that they have no competing interests.

## Authors’ contributions

YZ and ZS cooperated in the conception and design of the study and in the collection of the data. JW, HZ and ZL validated all pathology reports and assisted in data analysis and interpretation of data. ZS drafted the manuscript. All authors approved the final manuscript.
